# Complete Genome Sequence of an Oral Commensal, *Streptococcus oligofermentans* Strain AS 1.3089

**DOI:** 10.1128/genomeA.00353-13

**Published:** 2013-06-20

**Authors:** Huichun Tong, Nan Shang, Li Liu, Xinhui Wang, Jun Cai, Xiuzhu Dong

**Affiliations:** State Key Laboratory of Microbial Resources, Institute of Microbiology, Chinese Academy of Sciences, Beijing, Chinaa; Key Lab of Functional Dairy, College of Food Science and Nutritional Engineering, China Agricultural University, Beijing, Chinab; Network & Information Center, Institute of Microbiology, Chinese Academy of Sciences, Beijing, Chinac; University of Chinese Academy of Sciences, Beijing, Chinad

## Abstract

*Streptococcus oligofermentans*, an oral commensal, inhibits the growth of the dental caries pathogen *Streptococcus mutans* by producing large amounts of hydrogen peroxide. Therefore, it can be a potential probiotic for oral health. Here we report the complete genome sequence of *S. oligofermentans* strain AS 1.3089.

## GENOME ANNOUNCEMENT

*Streptococcus oligofermentans* was first described in 2003 (1). It is frequently isolated from healthy tooth surfaces of the human oral cavity (2). *S. oligofermentans* not only produces large amounts of hydrogen peroxide (H_2_O_2_), it also tolerates high concentrations of H_2_O_2_; these characteristics enable it to outcompete the dental caries pathogen *Streptococcus mutans* in a two-species biofilm model (3). It has been demonstrated that *S. oligofermentans* possesses multiple H_2_O_2_-generating enzymes, including lactate oxidase (Lox) (3), pyruvate oxidase (4), and l-amino acid oxidase (l-AAO) (5, 6). In particular, *S. oligofermentans* utilizes Lox to convert the abundant lactate produced by *S. mutans* into H_2_O_2_, which conversely inhibits the growth of *S. mutans* (3). Therefore, it has great potential to be developed into a probiotic for prevention of dental caries. The l-AAO-encoding gene of *S. oligofermentans* is acquired through horizontal gene transfer from other *Streptococcus* species (7). To better understand the interspecies interaction between *S. oligofermentans* and other oral streptococcus species in dental plaque and also their antioxidant defense mechanisms, we sequenced the complete genome of *S. oligofermentans* strain AS 1.3089.

The genome was sequenced at the University of Oklahoma Genome Sequencing Center, using a Roche 454 GS FLX sequencer. A total of 254,858 reads, comprising 62,111,758 bases (approximate 29-fold coverage of the genome), were obtained. All reads were assembled using GS *de novo* Assembler software, which generated 431 contigs ranging from 76 to 121,160 bp. Relationships of the contigs were determined by multiplex PCR (8). Gaps were filled by sequencing the PCR products using ABI 3730xl capillary sequencers, and low-quality regions of the genome were resequenced. The genome was annotated by the prokaryotic genome automatic annotation pipeline (PGAAP) provided by National Center for Biotechnology Information (NCBI). Gene predictions were done using a combination of GeneMark and Glimmer (9, 10). Ribosomal RNAs were predicted by Rfam models (11). Transfer RNAs were predicted by tRNAscan-SE (12). Annotation was executed by searching against all proteins from complete microbial genomes using BLAST.

The length of the genome is 2,142,100 bp, with G+C content of 42%. The average length of coding sequence is 884 bp. The genome encodes 2,094 predicted proteins and 50 tRNAs and has 4 copies of 5S-16S-23S rRNA genes. We compared this genome with the genome sequences of an oral commensal, *Streptococcus sanguinis* strain SK36, and the dental caries pathogen *S. mutans* strain UA159 and found that 654 protein genes are shared by all three of the species and 741 genes are shared by the oral commensals only, whereas only 29 genes are shared with *S. mutans*, which is much less than the 193 genes shared between *S. sanguinis* SK36 and *S. mutans* UA159 (13). There are 670 genes unique to *S. oligofermentans*. Detailed comparative analysis with the genomes of oral commensals and dental pathogens might deepen our understanding of the occurrence and development of dental caries.

### Nucleotide sequence accession number.

The complete genome sequence of *Streptococcus oligofermentans* AS 1.3089 has been deposited in GenBank under the accession number CP004409.
